# Design and Testing of an EHR-Integrated, Busulfan Pharmacokinetic Decision Support Tool for the Point-of-Care Clinician

**DOI:** 10.3389/fphar.2016.00065

**Published:** 2016-03-30

**Authors:** Susan M. Abdel-Rahman, Matthew L. Breitkreutz, Charlie Bi, Brett J. Matzuka, Jignesh Dalal, K. Leigh Casey, Uttam Garg, Sara Winkle, J. Steven Leeder, JeanAnn Breedlove, Brian Rivera

**Affiliations:** ^1^Division of Clinical Pharmacology, Toxicology, and Therapeutic Innovation, Children's Mercy HospitalKansas City, MO, USA; ^2^Department of Pediatrics, University of Missouri-Kansas City School of MedicineKansas City, MO, USA; ^3^Department of Information Systems, Children's Mercy HospitalKansas City, MO, USA; ^4^Division of Hematology/Oncology, Rainbow Babies and Children's Hospital, Case Western Reserve UniversityCleveland, OH, USA; ^5^Department of Pharmacy, Children's Mercy HospitalKansas City, MO, USA; ^6^Department of Laboratory Medicine, Children's Mercy HospitalKansas City, MO, USA

**Keywords:** software design, decision support, therapeutic drug monitoring, bone marrow transplant, usability testing

## Abstract

**Background:** Busulfan demonstrates a narrow therapeutic index for which clinicians routinely employ therapeutic drug monitoring (TDM). However, operationalizing TDM can be fraught with inefficiency. We developed and tested software encoding a clinical decision support tool (DST) that is embedded into our electronic health record (EHR) and designed to streamline the TDM process for our oncology partners.

**Methods:** Our development strategy was modeled based on the features associated with successful DSTs. An initial Requirements Analysis was performed to characterize tasks, information flow, user needs, and system requirements to enable push/pull from the EHR. Back-end development was coded based on the algorithm used when manually performing busulfan TDM. The code was independently validated in MATLAB using 10,000 simulated patient profiles. A 296-item heuristic checklist was used to guide design of the front-end user interface. Content experts and end-users (*n* = 28) were recruited to participate in traditional usability testing under an IRB approved protocol.

**Results:** Decision support software was developed to systematically walk the point-of-care clinician through the TDM process. The system is accessed through the EHR which transparently imports all of the requisite patient data. Data are visually inspected and then curve fit using a model-dependent approach. Quantitative goodness-of-fit are converted to single tachometer where “green” alerts the user that the model is strong, “yellow” signals caution and “red” indicates that there may be a problem with the fitting. Override features are embedded to permit application of a model-independent approach where appropriate. Simulations are performed to target a desired exposure or dose as entered by the clinician and the DST pushes the user approved recommendation back into the EHR. Usability testers were highly satisfied with our DST and quickly became proficient with the software.

**Conclusions:** With early and broad stake-holder engagement we developed a clinical DST for the non-pharmacologist. This tools affords our clinicians the ability to seamlessly transition from patient assessment, to pharmacokinetic modeling and simulation, and subsequent prescription order entry.

## Introduction

Busulfan is an antineoplastic agent commonly used as part of chemical regimens to prepare patients for bone marrow transplantation (BMT). It also reflects a drug with a narrow “therapeutic window” or range of concentrations within which the drug is both safe and effective. When the plasma concentrations fall outside of this “window,” patients are at risk for engraftment failure or life-threatening hepatotoxicity. Simply following recommended busulfan dosing guidelines does not ensure safety and efficacy for individual patients owing to the wide degree of inter-individual variability in busulfan disposition. To minimize patient risk and maximize therapeutic outcomes, many clinicians rely on therapeutic drug monitoring (TDM). In fact, TDM-guided busulfan dosage adjustment is addressed in the U.S. Food and Drug Administration-approved busulfan product label [Busulfex® (busulfan), 2007]. However, operationalizing the TDM process can be fraught with inefficiency.

We examined the TDM process at our institution and discovered that much of the inefficiency we observed could be attributed to outsourcing of the busulfan plasma concentration analysis and the related modeling and simulation (Table 1). Apart from the inefficiencies this introduced into care, we also had concerns about the quality of care we were delivering. In the pharmacokinetic reports provided by the reference lab, we observed inaccurate assumptions being made about individual plasma concentrations, false suppositions related to sample timing, and the application of inappropriate mathematical models to describe our patients' data. The consequence was an overestimation of the dose needed to achieve the desired target exposure in nearly 100% of the patients we were treating.

**Table 1:** **Inefficiencies in the busulfan TDM process at our institution**.

**Inefficiencies**	**Details**
Restricted scheduling	• Owing to limited availability of RL personnel, scheduling of BMT patients was restricted to the first 4 days of the week
Time intensive preparatory activities	• No less than 48 h before the TDM study, BMT staff must identify the ordering physician and the physician who will be receiving the results, obtain hard copy signatures from both, and transmit both electronic *and* verbal orders to the RL
	• The team must also coordinate specimen collection and non-routine shipping for the in-house lab
Redundant pre-delivery activities	• Information related to dosing and specimen collection is documented in EHR and transcribed onto a separate hard copy requisition form duplicating activities and introducing the potential for error
	• Transmittal of these forms relies on the availability of a BMT team member and introduces unnecessary delays while the responsible party is attending to other clinical duties
Inflexible PK analysis	• Remote modeling and simulation requires interruption of the physician's workflow to accommodate a verbal call from the PK specialist
	• Further, the lack of access to the modeling process limits the ability of the BMT team to refine the mathematical approach, and affords the team no flexibility to examine alternative dosing strategies, should relevant clinical information arise after the requisition has closed
	• Finally, the need relay dosing recommendations by phone and fax does not permit seamless entry of the new orders into the EHR

*BMT, bone marrow transplantation; EHR, electronic health record; PK, pharmacokinetic; RL, reference laboratory; TDM therapeutic drug monitoring*.

We were able to eliminate selected inefficiencies by transitioning busulfan specimen analysis from the reference lab to our internal Clinical Toxicology laboratory (Deng et al., 2016). We also considered an automated consult to the Clinical Pharmacology service to mitigate our mathematical concerns; however, implementation of a consultation process would not address efficiency issues. We recognized that providing the point-of-care clinician with training and access to external TDM software was infeasible. Not only would this strategy interrupt physician workflow, these tools can be unnecessarily complex to navigate and are rarely designed with the physician user in mind (Barrett, 2015). Instead, we chose to develop a unique decision support tool aimed at the clinician and embedded in our electronic health record (EHR). This manuscript details the development and testing of a clinical decision support tool created at our institution for use by our BMT colleagues.

## Materials and methods

### Design

Our development strategy was largely modeled based upon the published features associated with successful decision support tools (DSTs; Lobach et al., 2012). These include (1) involvement of local users in the development, (2) integration within the charting/order entry system, (3) availability at the time and location of decision making, (4) avoidance of a requirement for additional data entry, (5) provision of a recommendation as opposed to an assessment, and (6) justification of the decisions with evidence.

### Stake-holder engagement

The project began with a Requirements Analysis to assure a basic understanding of user needs and the requirements necessary for optimal TDM execution. The existing process was analyzed and the sequence of tasks along with information flows were documented. High-level tasks were broken down into subtasks and operations, and the informational sources required in each subtask were recorded. The output of the requirements analysis included process charts, task flow diagrams, task decomposition tables, and use case scenarios. Understanding this workflow was critical to inform development of the application. By engaging physicians, nurses, pharmacists, pharmacologists, and clinical laboratory personnel we were able to draw perspectives from the various providers that are (or would soon be) involved in the TDM process.

### Back-end development

The software flow and nested logic used to code the modeling and simulation components of the software were based on the algorithm established by the clinical pharmacologist who manually performed the modeling and simulation after transitioning busulfan analysis to our institutional laboratory (Abdel-Rahman et al., 2016). We formulated our compartmental pharmacokinetic modeling approach as a non-linear least square problem, solved by the Levenberg-Marquardt algorithm with initial parameters estimated by curve-stripping. A series of nested logic functions were used to determine whether the data should be fit to a one- or a two-compartment model. Quantitative goodness-of-fit (GOF) criteria, traditionally used to determine the appropriateness of a model (e.g., weighted sum of squares, coefficients of variation the coefficients and exponents), were log-adjusted, weighted, and combined to generate a 10-point scale that is overlaid on a visual indicator alerting the user to the strength of the model. The equations for a non-compartmental model were also coded for implementation under certain criteria and/or with end-user override of the compartmental analysis. Ten-thousand patient profiles were simulated to test each executable path and the output validated against the findings generated by applying the same calculations in MATLAB®. The algorithm was initially implemented in portable standard C++ language as the computing core of the project and subsequently converted to C# to facilitate integration with the user interface (UI).

### Front-end design

Using the information gathered from the Requirements Analysis, a wireframe prototype of the application to test the DST's functionality was developed in Visual Studio. A web-design expert wrote the necessary code to integrate the back-end code with the front-end UI and designed the UI for optimal functionality on both desktop and portable devices using Chrome and Internet Explorer browsers. Javascript and Javascript libraries such as jQuery, jQueryUI, JSON, and the Highcharts visualization package were used heavily in the development of the UI to achieve a consistent experience across devices and web browsers. A 296-item heuristic checklist was used to guide design of the front-end UI (Weiss, 1993). Major areas of focus included: Visibility of System Status, Match Between System and the Real World, User Control and Freedom, Consistency and Standards, Error Recognition/Prevention/Recovery, Flexibility and Minimalist Design, Aesthetic and Minimalist Design, Help and Documentation, Interaction with the User, and Privacy. The UI was iteratively refined after usability testing.

### Integration with the EHR

To maximize design flexibility and minimize restricting our application to a single EHR system, we elected to integrate our software as link within the EHR (currently operating using Cerner®). We developed a PowerForm that centralizes the multidisciplinary activities associated with busulfan TDM. Ordering a busulfan TDM study prompts a “face up” view of the PowerForm that generates all tasks and queries related to the study. A static order set generates labels for specimen collection. Data holding spots were created for entry by pharmacy (dosing weight, study dose number, total number of doses) and nursing (infusion start/stop times, sampling times, catheters accessed for dosing and sampling). Indicators signal the completion of all necessary tasks. The PowerForm serves to facilitate coordination of the TDM study and eliminates duplicative data capture. Preparatory and pre-delivery activities were streamlined and the steps where transcription errors could be introduced were eliminated addressing additional inefficiencies enumerated in Table 1.

Clicking on the software link within the PowerForm transparently pulls in all of the relevant patient data from the PowerForm, along with demographic data, clinical laboratory data, and dosing information from the medication administration record. Accessing the software outside of the EHR requires provision of a medical record number which then permits import of the same data described above. The software was also built with a manual entry feature to accommodate the analysis of data from patients that are not contained within our institution's EHR. Completion of the modeling and simulation within the DST pushes a time/date stamped report back into the EHR while completion of more than one report for the same child prompts a warning indicating that a report has already been submitted for that child. We elected not to integrate software recommendations with computerized physician order entry (CPOE) until the software has been prospectively validated.

### Usability testing

Structured cognitive walkthroughs (CW) were conducted with individuals representing medicine, pharmacy, pharmacology, and nursing to identify whether the interface supported the prospective users' needs (Polson et al., 1992). Recommendations arising from the CW were incorporated into the application's design. Subsequently, 14 content experts (CE) and 14 end-users (EU) participated in traditional usability testing to assess efficiency, ease-of-use, and user satisfaction with the application among a representative user populations (Table 2). Content experts reflected individuals having completed formal post-doctoral training in Pediatric Clinical Pharmacology. End-users reflected clinicians and/or administrative staff at the hospital that could reasonably be expected to interact with the software. Participants were observed as they completed a series of tasks within the application centered around four distinct clinical scenarios. Testing incorporated a think-aloud protocol in which participants were encouraged to verbalize what they are thinking during testing. Audio and video of the participants' interaction with the DST were captured using logging software designed for usability testing (Techsmith Morae). Additional information was collected via questionnaire. Each usability test gathered the following measures: demographics, time to complete tasks, number of clicks required to complete tasks (compared to optimal paths), task success, and user perceptions of task ease/difficulty. Overall user satisfaction and perceived usability was measured using version 3 of the Post-Study System Usability Questionnaire (PSSUQ v3; Sauro and Lewis, 2012). Standard descriptive statistics were applied to describe the usability testing participants and the PSSUQ response data. Statistical differences in task performance between user subpopulations were examined by application of a two-tailed, unpaired Student's *t*-test. The significance limit accepted for all statistical analyses was α = 0.05. All participants were enrolled with informed consent under a protocol that was reviewed and approved by the Institutional Review Board at Children's Mercy Hospital.

**Table 2:** **Characteristics of participants in the usability testing**.

**Characteristic**	**Group**	**Content experts (*n* = 14)**	**End users (*n* = 14)**
Gender	Male:Female	6:8	3:11
Age group (year)	26–39:40–59	8:6	11:3
Race	Afr. American:Asian:Caucasian	2:2:10	0:2:12
Current role	Resident/Fellow	1	0
	Physician	11	3
	Pharmacist	1	7
	Pharmacologist	1	0
	Nurse	0	2
	Administrator	0	2
Years in current role	< 5:5–10:10–15:15± years	5:4:1:4	7:7:0:0
activities performed on the computer aside from email	median (range)	5 (2–6)	5 (3–7)
Hours per week spent on a computer	5–15:15–25:26±	1:3:10	0:3:11
Computer platform most often used	Mac:Windows:Both	0:12:2	4:6:4
Different browsers used for computing	IE:Chrome:Firefox:multiple	2:4:0:8	1:5:1:7
Frequency with which an EHR is accessed	Daily	10	11
	Weekly	2	1
	Monthly	1	0
	A few time a year	0	0
	Never	1	2
Frequency with which a computerized clinical decision support tool is used to assist in patient management	Daily	0	1
	Once or twice a week	3	2
	About once a month	3	8
	A couple of times	2	1
	Never	6	2
Frequency with which TDM is used to influence clinical decision making	Daily	0	2
	Once or twice a week	3	5
	About once a month	5	1
	A couple of times	4	2
	Never	2	4
Proficiency with pharmacokinetic calculations	Strong	0	2
	Moderate	10	5
	Weak	3	2
	Not proficient at all	1	5
Frequency with which PK calculations are applied to patient care	Daily	0	0
	Once or twice a week	0	4
	About once a month	2	2
	A couple of times	7	0
	Never	5	8
Tool most often used for PK calculations applied to direct patient care	Handheld calculator	3	6
	Microsoft Excel	6	0
	Commercially available software	0	0
	N/A	5	8

## Results

The DST we developed systematically walks the end-user through a series of screens that select the most robust mathematical model around which to perform simulations that target user-defined inputs. In total, the user can arrive at a new dose and push the clinical decision back to EHR in four mouse clicks; however, there are options for additional decision making at each screen.

**Figure 1:**
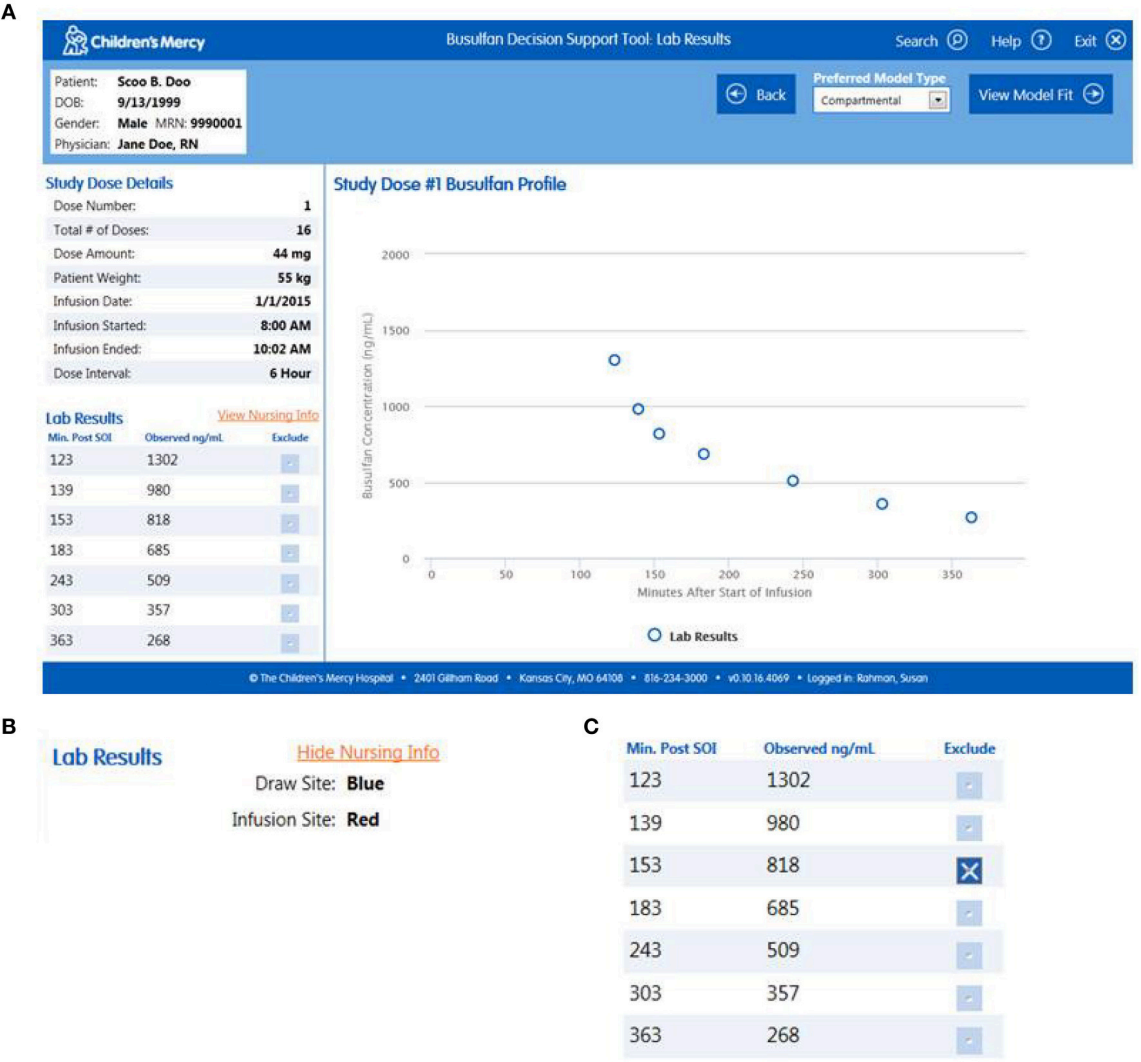
**(A)** “Lab Results” page of the software with expanded views of the fields that allow the user to **(B)** view nursing notes, and **(C)** exclude data points.

**Figure 2:**
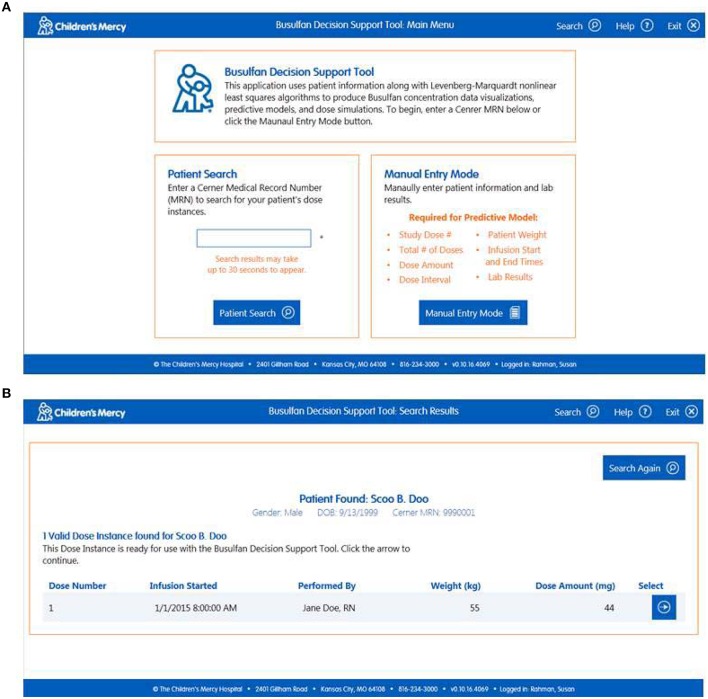
**(A)** “Main Menu” page visible when users access the software outside of the EHR. **(B)** “Search Results” page that permits users to select the TDM study of interest.

**Figure 3:**
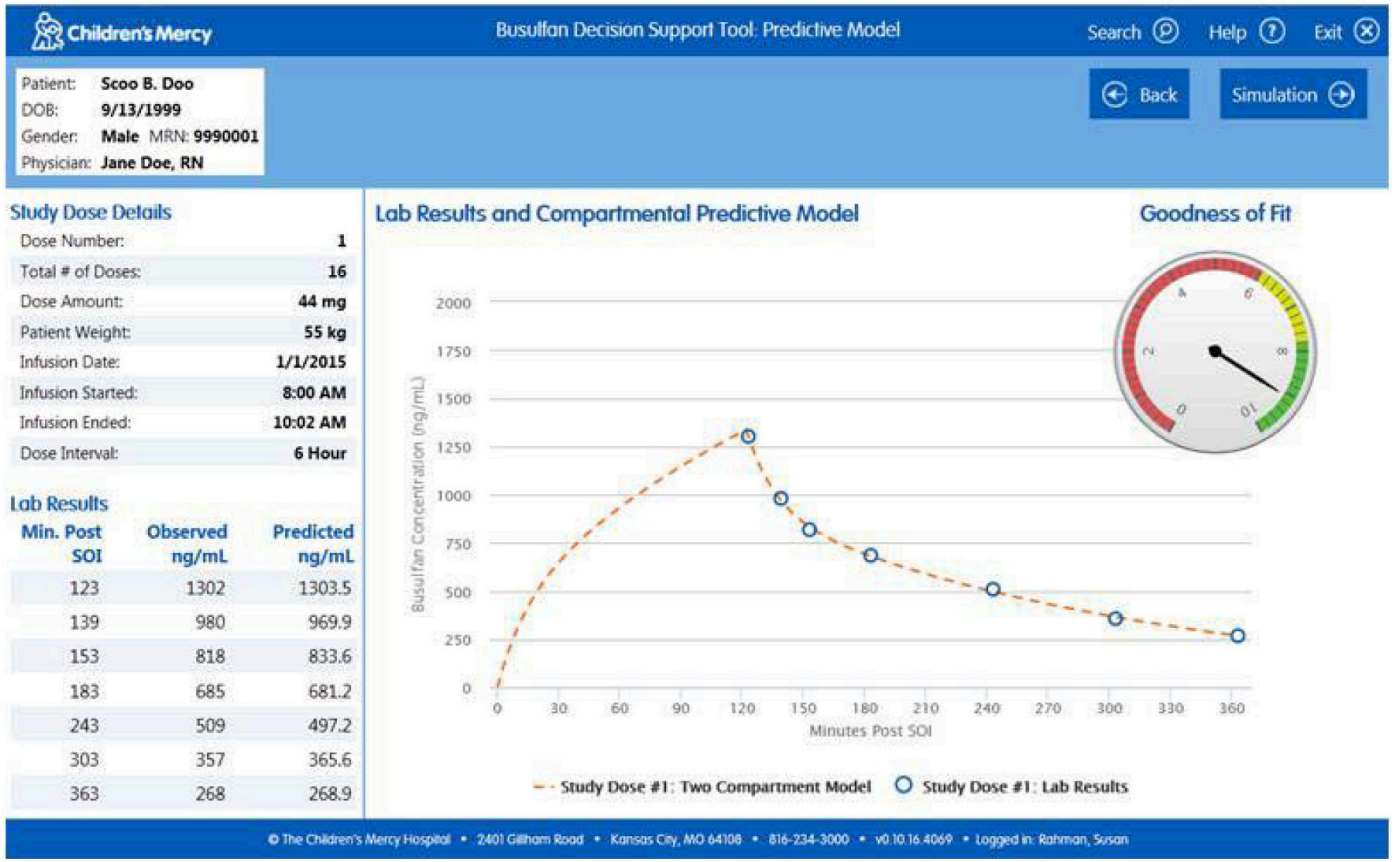
**“Predictive Model” page of the software on which on which the user can examine the appropriateness of the default or selected model**.

**Figure 4:**
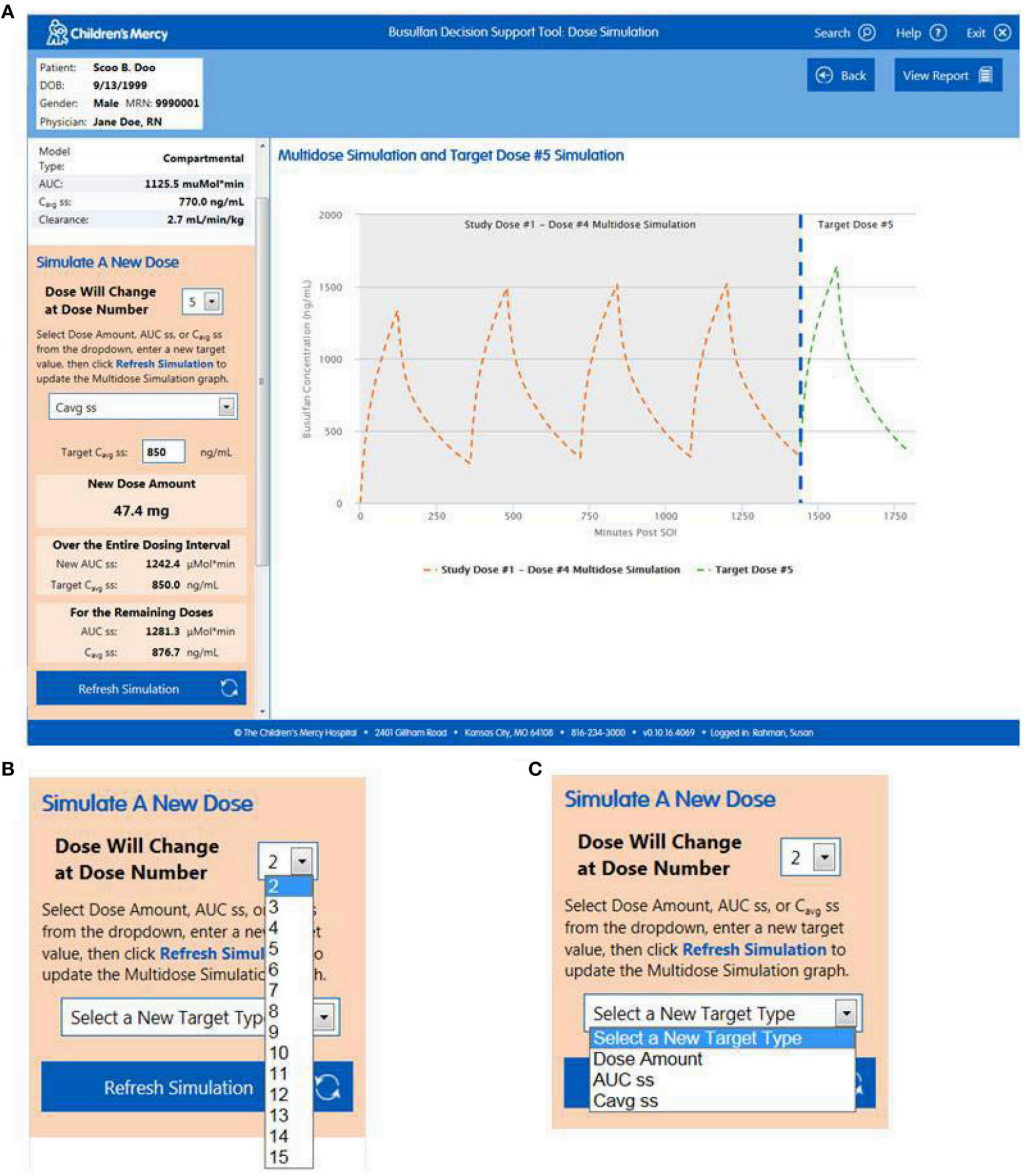
**(A)** “Dose Simulation' page of the software from which users can conduct simulations for target dose or exposure values. Dose change **(B)** and target **(C)** drop down menus are detailed.

**Figure 5:**
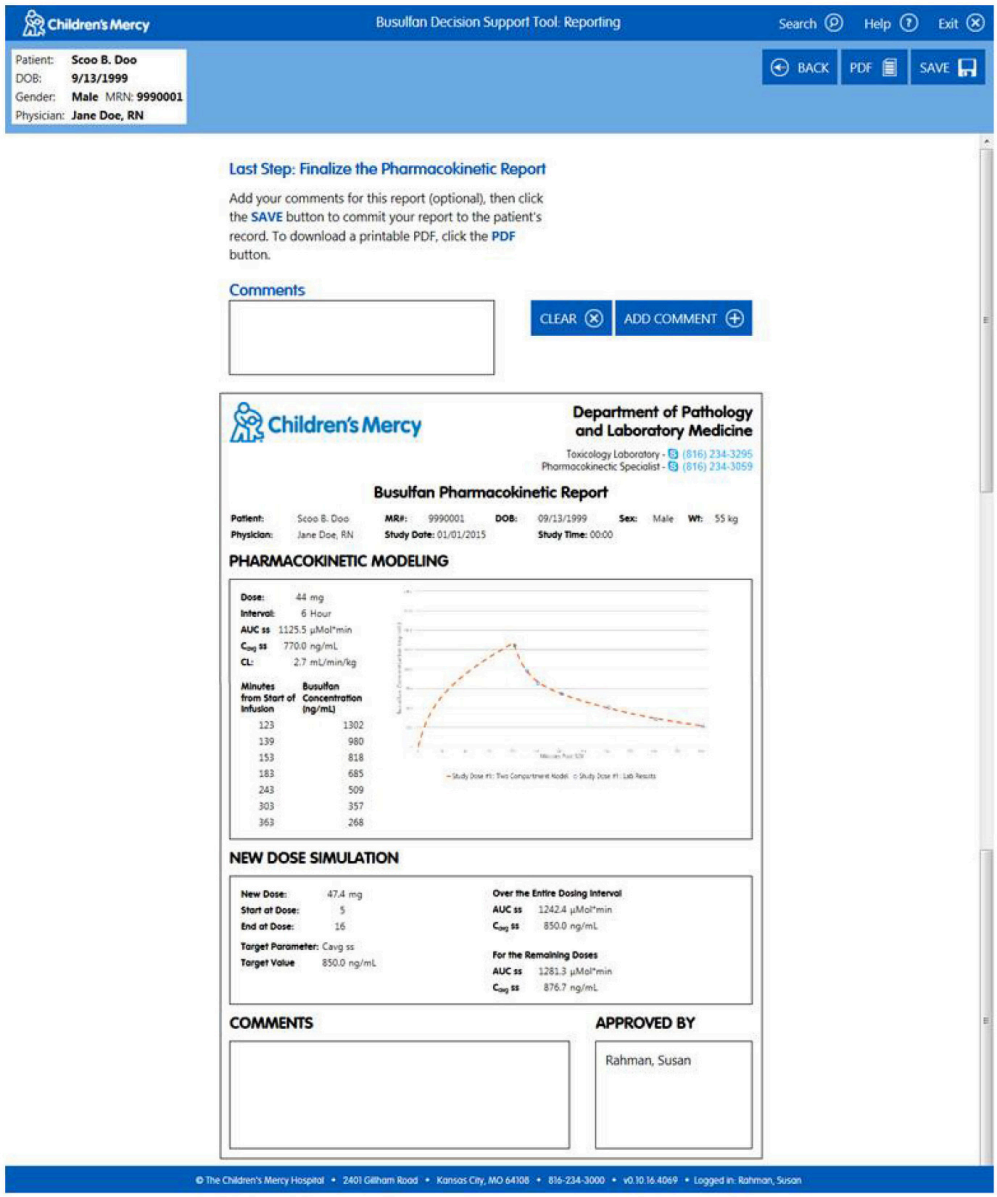
**“Reporting” page of the software where end users can finalize their recommendations and push the information back to the EHR**.

1. *Import Patient Data*. Clinicians can access the software within the EHR or externally on the hospital's server. When accessed via the patient's medical record the user arrives at the “Lab Results” page which permits them to confirm the patient information and visually inspect the data (Figure 1A). All of the relevant information needed to perform the analyses (e.g., dose, infusion duration, sample times, etc.) are imported transparently from the EHR. The user can view the nursing notes on this page (Figure 1B) and, if relevant, exclude data they believe to be erroneous or contaminated (Figure 1C). If users access the software external to the EHR, they arrive at a screen that allows them to input a medical record number or select manual entry (Figure 2A). Entering a medical record will pull up all instances of a busulfan TDM study for that child from which the user can select the relevant dataset (Figure 2B).
2. *Perform Curve Fitting*. The only executable feature on the “Lab Results” page is a “View Model Fit” button. The default model is a compartmental model but the user can override this by selecting a non-compartmental model. The software will also transparently default to a model-independent approach when selected criteria are met. Executing “View Model Fit” takes the user to the “Predictive Model” page where they will see a graphic of the curve fit and the model-type that was fit (Figure 3). Additional features allow the user to examine the goodness-of-fit including concentrations predicted by the model which lay adjacent to the actual patient values and a tachometer which consolidates the goodness-of-fit criteria discussed above into a single indicator (Figure 3). “Green” alerts the user that the model is strong while “yellow” signals caution and “red” indicates that there may be a problem with the fitting. Users that are satisfied with the model can proceed to simulation. If the user is dissatisfied with the model they can go back to the “Lab Results” page, re-inspect the raw data to determine whether selected samples need to be excluded from the fit, and/or decide whether they should override the default fitting strategy to perform model-independent analyses.
3. *Perform Simulations*. On the “Dose Simulation” page users will see the patient's clearance value and predicted exposure levels (i.e., AUC and Cavg ss) with sustained administration at the initial dose (Figure 4A). Users can enter the dose number at which the regimen will be changed (Figure 4B) and the therapeutic target around which the simulations will be based (Figure 4C) along with the numeric value for that target. The simulations can be repeated ad infinitum to examine the recommended dose for different exposure targets or the resultant exposure values if the dose were rounded up/down.
4. *Finalize the Report*. When users have decided on a final recommended dose-exposure combination, the “View Report” button will take them to the final “Reporting” page where they can review the recommendation, add comments, print the report (if desired), and push the report back into the EHR (Figure 5). The report is displayed in, and can be retrieved from, both the “Results Review” tab and the “Documents” section of the EHR.

Usability testing revealed that our DST could be efficiently navigated by our testers. Median task time across all tasks was 9.6 s with a median 1.6 mouse clicks per task. Greater than ninety-seven percentage of tasks were completed with no difficulty. When selected tasks were encountered more than once, task time significantly dropped (Table 3). On average, EU completed tasks slightly faster than CE (14.9 vs. 18.4 s); however, this was only significant for the tasks associated with the second (8.3 vs. 11.5 s, *p* = 0.05) and third (11.6 vs. 15.3 s, *p* = 0.01) clinical cases presented to the testers. With respect to the remaining background characteristics, performance metrics were largely uniform across our population. Observable demographic difference in performance metrics could be noted for females who completed all assigned tasks across all four cases with fewer number of clicks than males (79.8 vs. 96.2, *p* = 0.03) and for participants 26–39 years of age who demonstrated total task times (465 vs. 729 s, *p* = 0.01) and average task times (14.1 vs. 22.1 s, *p* = 0.01) that were shorter than participants aged 40–59 years.

**Table 3:** **Time in seconds to complete selected tasks**.

**Task**	**Event #1**	**Event #2**	**Event #3**	**Event #4**
Import data^a^	31.1 ± 15.5	15.7 ± 8.3	11.6 ± 9.8	10.9 ± 6.8
Inspect the data^a^	26.8 ± 18.8	11.1 ± 6.9	8.9 ± 7.1	10.9 ± 7.2
Perform curve fitting^b^, ^c^	18.5 ± 13.6	4.3 ± 4.9		
w/NCA warning introduced			12.7 ± 4.2	7.2 ± 6.4
Evaluate the mathematical goodness-of-fit^b^	14.3 ± 15.0	8.3 ± 17.5		
Identify the type of model that was fit^b^	19.0 ± 16.7	3.5 ± 2.1		
Perform simulation to achieve a specified therapeutic target^d^	77.3 ± 135.0	27.3 ± 22.4	19.0 ± 9.9	
Identify the new dose	5.9 ± 8.7	3.3 ± 2.1		
Examine the exposures with the modified dose^b^	14.7 ± 14.4	8.6 ± 5.3	11.1 ± 7.4	
Finalize the report	5.7 ± 4.7	6.9 ± 6.3	4.0 ± 3.5	

*Data are represented as mean ± standard deviation. NCA, non-compartmental analysis*.

a *Events #2, #3, and #4 significantly faster than event #1 (p < 0.01)*.

b *Event #2 significantly faster than event #1 (p < 0.01)*.

c *Event #4 significantly faster than event #3 (p < 0.01)*.

d *Events #2 and #3 significantly faster than event #1 (p < 0.05)*.

**Figure 6:**
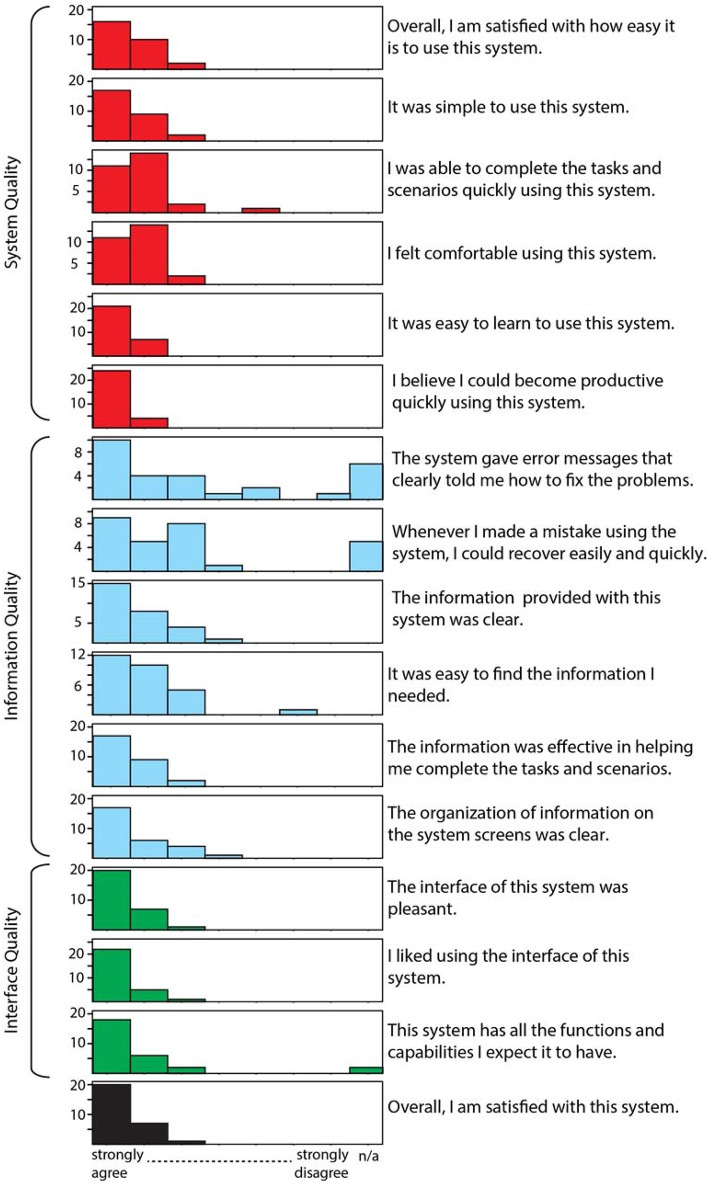
**Histogram of PSSUQ responses provided by our usability testers**.

On a 7-point Likert scale where 1 represents the most favorable response and 7 the least favorable response, overall satisfaction rated a 1.55; with scores of 1.47 for system quality, 1.81 for information quality, and 1.32 for interface quality. The distribution of scores for each element of the PSSUQ is provided in Figure 6.

## Discussion

By virtue of its complexity, TDM represents a multi-disciplinary approach to disease management. Successful application of TDM to patient care requires (1) a comprehensive knowledge of the patient, (2) a thorough understanding of the pharmacologic principles that drive the relationship between dose-exposure-response, (3) expertise in mathematical and pharmacokinetic (PK) modeling and simulation, and (4) precise and accurately recorded specimen collection (Durieux et al., 2008). However, TDM-related activities can be relatively arduous and are, therefore, commonly relegated to a PK specialist who may have no regular involvement with the patient and their care team (i.e., TDM activities are external to the normal flow of care; Buclin et al., 2012).

Attempts to integrate TDM into the clinical care work flow have been pursued for over three decades. Countless computer-based tools that embed various functionalities have been developed (Sheiner et al., 1972; Proost and Meijer, 1992; Buffington et al., 1993; Lacarelle et al., 1994). The vast majority exist on standalone computer systems and only a few were designed to interface with the electronic health record (Nieuwlaat et al., 2011; Fuchs et al., 2013). Though the intended target user for many of these systems has been the physician, less than half underwent any pilot testing and for those systems where studies were undertaken, performance was evaluated in the hands of a PK specialist rather than the non-specialized end user (Nieuwlaat et al., 2011). Independent evaluations looking at the usefulness and usability of these computerized tools have also engaged pharmacists and clinical pharmacologists who already possess expertise in TDM (Fuchs et al., 2013). Importantly, essentially none of these tools have experienced distribution beyond the academic center at which the software was developed. As a result, many of these systems have failed to reach a broader audience and, thus, have failed to demonstrate improvements in the process of care or in patient outcomes (Nieuwlaat et al., 2011).

We designed a software tool that integrates the multidisciplinary activities involved with busulfan TDM in an attempt to enhance process efficiency and improve satisfaction of our BMT team with TDM-driven busulfan dosing decisions. The tool we developed differs markedly from the evolution of nearly all hospital-based TDM services where Pharmacy or Clinical Pharmacology is consulted and queued into the patient care process. We have no knowledge of institutions wherein the modeling tool we describe has been successfully placed into the hands of the provider. In fact, the reverse is often seen wherein the modeling and simulation process is presented as a “black box” to protect the professional activities and billable domain of the pharmacokientic specialist (Neely and Jelliffe, 2008). However, a focus on reimbursement for a singular department fails to consider the overall cost savings that may be appreciated when the efficiency of the entire system is improved.

The novel DST that we developed affords providers far more flexibility when it comes to caring for patients that require TDM-guided dose optimization than traditional TDM practice models. The software can be accessed 24 h a day, 7 days a week from any computer with access to the EHR. This feature lifts restrictions as to when the TDM studies can be performed and enables clinicians to repeat simulations at any time during the patient's course of therapy. Since the user never leaves the EHR, the transition from dose simulation to order entry is seamless. This approach nests numerous features associated with successful DSTs, (Lobach et al., 2012) and addresses several of the inefficiencies enumerated in Table 1. Importantly, Clinical Pharmacologists familiar with pharmacokinetic modeling and simulation remain accessible to the BMT team by pager; however, the clinical team members for whom the patient is their primary responsibility are now the drivers of the TDM activities.

The effective TDM-based DST should be designed and vetted by a multi-disciplinary team to ensure that it performs optimally and supports the needs of the end-users. It should also integrate directly into the EHR and intelligently filter, organize, and deliver user-specific information at appropriate times to maximize the efficiency and quality of care that the patient receives (Fuchs et al., 2013). Both of these criteria were met in the design and development of our DST as evidenced by the objective performance metrics and subjective scores assigned by our testers. As expected, testing in a broad range of users revealed several changes that our users would like to see integrated into the tool and the software is currently being revised to incorporate these recommendations. A possible limitation of our testing is that the EU comments were generated from BMT team members working together at a single institution who may share a similar understanding of TDM. Soliciting additional feedback from a broader test audience outside of our institution may reveal additional elements of the DST that require modification.

Immediate next-steps relate to quality assurance and involve prospectively examining the accuracy and predictive performance of the DST's back-end processing algorithm. Notably, very few investigations to date have examined whether the standard-of-care predictions as performed in clinical reference laboratories are corroborated by observed busulfan levels after dose adjustment. In addition, we would like to evaluate whether the steady-state busulfan concentrations predicted by the DST software are corroborated by the actual concentrations observed in BMT patients. These activities will expose whether the back-end model can be augmented by the inclusion of (as of yet undescribed) patient-specific factors that may influence the accumulation of busulfan at steady-state. Additional future considerations include deployment of the tool beyond our institution. Our decision to build a DST that can integrate into commercially available EHR software was driven by the goal of making the tool accessible beyond our institution. Deployment with partnering hospitals is in discussion and will be considered after a satisfactory period of quality assurance and receipt of the necessary regulatory approvals.

## Author contributions

SA conceived of the application, developed the algorithm around which the software was based, led the development of the decision support tool, and conducted the usability testing; JD and KC coordinated the requirements analysis for the clinical aspects of this tool; UG coordinated the requirements analysis for the laboratory components in this tool; SW was responsible for building the necessary supporting elements within our electronic health record; CB was involved in coding of the back-end analytics; BM undertook unit testing and validation; MB was responsible for the design and coding of the user interface; BR was responsible for supervising all informatics activities and for integration of the software with the electronic health record; JL and JB facilitated institutional implementation of the software. All authors reviewed and approved the manuscript.

## Funding

Funding for the development of this software was provided, in part, by a gift from the Victor E. Speas Foundation-Bank of America, Trustee.

### Conflict of interest statement

The authors declare that the research was conducted in the absence of any commercial or financial relationships that could be construed as a potential conflict of interest.
